# The Evaluation of Zinc Source and Dose in Broiler Diets Fed at Different Calcium and Phosphorus Levels in the Presence or Absence of Phytase

**DOI:** 10.3390/ani16162485

**Published:** 2026-08-10

**Authors:** Jamie L. Fourie, Kyle M. Venter, Peter W. Plumstead, Christine Jansen van Rensburg, Clara R. Angel, Cibele A. Torres

**Affiliations:** 1 Neuro Livestock Research, Kameeldrift, Brits 0250, South Africa; jamie@nlrhub.com (J.L.F.); peter@nlrhub.com (P.W.P.); 2 Department of Animal Sciences, Stellenbosch University, Stellenbosch 7600, South Africa; christinejvr@sun.ac.za; 3 Department of Animal Science, University of Pretoria, Pretoria 0002, South Africa; 4 Department of Animal and Avian Sciences, University of Maryland, College Park, MD 20742, USA; rangel@umd.edu; 5 Zinpro Corporation, 5831 PJ Boxmeer, The Netherlands; ctorres@zinpro.com

**Keywords:** broiler, organic zinc, inorganic zinc, calcium, phosphorus

## Abstract

Phytate is a strong chelator of macro- and microminerals in the gastrointestinal tract. Under an environment in which phytate is rapidly hydrolyzed, the digestion and absorption of Ca, P, Zn and other cations from raw materials can be increased. This study investigated the performance, mineral excretion and bone mineralization of broilers fed diets formulated to optimize phytate hydrolysis and supplemented with Zn in the form of a Zn amino acid complex (Zn-AA) or as Zn sulfate (Zn-S). A total of 5040 day-old Ross 308 straight-run broiler chicks were allocated to 84 pens in a complete randomized block design, with 7 treatments and 12 replicates per treatment. Treatments 1 and 2 followed the breed recommendations (RECs) for Ca and P inclusion according to a three-phase feeding program and were supplemented with 90 ppm Zn as Zn-S, with (treatment 1) or without phytase (treatment 2), where treatment 1 is representative of a typical commercial-type diet. The remaining five treatments followed the University of Maryland (UMD) REC for Ca and P inclusion in a five-phase feeding program. The UMD diets were supplemented with either 90 ppm Zn-S (treatment 3) or graded levels of Zn-AA at 20, 40, 60, and 80 ppm (treatments 4–7). Phytase (2000 FTU/kg) was added to all diets except treatment 2. Data were analyzed in JMP 16.0 using one-way ANOVA regression analysis for Zn-AA treatments and Rao–Scott chi-square analysis for categorical data. Broilers fed 80 ppm Zn-AA resulted in heavier (*p* < 0.05) bodyweight (BW) on d 10, 16, 24, 30, and 37 when compared to all birds fed Zn-S at 90 ppm. On d 10, broilers fed 80 ppm Zn-AA had a higher (*p* < 0.05) tibia ash and tibia Zn content compared to those fed 90 ppm Zn-S (285.34 vs. 258.53 mg/tibia; and 12.00 vs. 10.62 mg Zn). Breast yield was also higher when comparing 80 ppm Zn-AA and 90 ppm Zn-S using the UMD REC (30.12 vs. 29.32%). Furthermore, broilers fed 40 ppm and above of organic Zn had less severe back scratches at term. It was therefore concluded that supplementation of Zn-AA following the UMD Ca and P REC resulted in increased growth performance and promoted higher breast yield of broilers whilst improving welfare.

## Simple Summary

Zinc (Zn) is an essential mineral commonly added to broiler diets to support growth, immunity, and skin health. However, the current zinc recommendations were established years ago in the absence of phytase. Phytase is now routinely included in poultry diets to improve the availability of nutrients, including Zn. This raises the question of whether existing Zn supplementation levels are still relevant. Furthermore, calcium (Ca) and phosphorus (P) can interact with Zn and may affect its utilization by broilers. Another important factor is the source of Zn, as Zn complexed to an amino acid is often considered to be more available than inorganic Zn sources. In addition to its role in growth and mineral metabolism, Zn is essential for wound healing and maintaining skin integrity, suggesting that Zn nutrition may also influence the incidence and severity of skin lesions such as back scratches, an important indicator of broiler welfare. This study aimed to evaluate the effect of organic Zn supplementation at various doses on performance, mineral excretion, bone mineralization, and back scratches in broilers, in comparison to the Zn supplementation strategies typically used by the commercial industry, as well as the Ca and P recommendation used in commercial nutrition. Additionally, this study assessed the optimal levels of zinc when complexed to amino acids as supplemented in diets with phytase. The results showed that the use of organic forms of Zn improved broiler growth and breast meat yield compared to inorganic Zn (zinc sulfate) fed at a higher dose. Birds fed organic Zn also had fewer and less severe back scratches, indicating improved welfare. These findings suggest that optimizing both the source and level of Zn, while considering dietary Ca and P levels, can enhance production efficiency and support animal welfare in modern broiler systems.

## 1. Introduction

Zinc has multiple pivotal roles in broiler physiology, including musculoskeletal development, immunity, cellular signaling, enzymatic functions, DNA synthesis, and feathering [1,2]. It is evident that Zn is an essential mineral in the avian body, with deficiencies resulting in an impaired physiological state and detriments to subsequent growth. In commercial broiler diets, Zn is typically supplemented in inorganic forms, such as Zn oxide (ZnO) or Zn sulfate (Zn-S). Due to the lower bioavailability of inorganic Zn [3,4,5,6], production systems tend to include Zn at high levels of around 120 ppm in a commercial broiler diet [7,8]. However, there has been recent pressure to reduce mineral excretion from intensive poultry production systems due to the effect of minerals on soil accumulation, leeching, and ecotoxicity [9,10]. This highlights the need to re-evaluate Zn supplementation levels in modern broiler nutrition.

High inclusion levels may lead to mineral antagonism and increased environmental excretion. Inorganic Zn quickly dissociates in the acidic conditions of the proventriculus and gizzard, increasing the quantity of soluble and thus reactive (charged) trace minerals accessible in the upper gastrointestinal tract (GIT). This increases the likelihood of their binding with phytate to form insoluble complexes, decreasing the availability of Zn for absorption [11,12,13,14,15] and increasing mineral excretion into the environment.

In contrast, organic forms of Zn are typically chelated to organic ligands such as amino acids, preventing rapid dissociation in the upper GIT. This reduces interactions with phytate and improves the bioavailability of minerals to the bird [5,6]. The nature of the ligand used in the various organic Zn products may contribute to inconsistent findings in the literature. While several studies have reported improved growth performance with organic Zn supplementation [16,17,18], others observed no significant differences in growth between broilers fed diets containing different Zn sources [19,20,21]. The variability among study outcomes highlights the importance of considering the ligand associated with Zn when evaluating its effect on the response of the broiler and biological efficacy.

The interpretation of Zn supplementation studies is further complicated by the widespread use of exogenous phytase in modern poultry diets. Poultry diets are commonly supplemented with doses of phytase which aim to completely dephosphorylate phytic acid, which is the main storage form of phosphorus (P) in plant seed-based feed ingredients. In addition to improving P availability, phytase exhibits a wide range of extra-phosphoric effects in the broiler [22]. This is, in part, due to the propensity of phytate to chelate with divalent cations [23], decreasing mineral availability to the broiler. Given that two of the most potent mineral chelators to phytate are Zn and Cu [23,24], the use of a highly efficacious phytase at high doses would aid in liberating the Zn in the raw materials of the diet, which is typically around 30 ppm [25,26]. Furthermore, recent advances in phytase efficacy have raised expectations of near-complete phytate degradation under commercial conditions [27]. Additionally, due to the tendency of calcium (Ca) to chelate with phytate, and this interaction with Zn [11], it may be worthwhile to investigate if diets formulated under different Ca and P recommendations (RECs) affected the responses in broilers when fed the same dose and source of Zn in conjunction with a high dose of phytase. Recent nutritional strategies have focused on aligning mineral supplementation with the physiological requirements of broilers to reduce excess mineral excretion. For example, recommendations from the University of Maryland (UMD) have been developed with the goal of more accurately meeting the broilers’ Ca and P requirements to optimize Ca and P nutrition. In the UMD REC, there is a focus on supporting skeletal development during the first 16 d of life through providing a slightly higher Ca and P content in the pre-starter diet, followed by the removal of inorganic P supplementation after d 16 (given that a high level of an efficacious phytase is used). This approach decreases the use of inorganic phosphates, which are both costly and highly polluting [28].

Few studies have been done to determine the effects of a high dose of modern phytase (where one may expect a high degree of phytate degradation) on the response of broilers to Zn supplementation. Furthermore, to the authors’ knowledge, no estimation of the Zn requirements in diets that have high phytase supplementation has been reported. This may affect the response of the broilers to different Zn sources, including organic forms such as Zn-AA. It is therefore important to reassess the level of Zn supplementation needed under conditions that reflect current industry practices, including the use of highly efficient phytases [27]. Therefore, the present study aimed to (1) evaluate the effects of organic Zn supplementation at various doses on broiler performance, skin integrity, and bone mineralization in comparison to broilers fed diets with the Zn source and dose commonly used in commercial industry; (2) determine the efficacy of graded inclusion levels of an organic Zn product as the sole Zn source in phytase-supplemented diets; (3) assess the effect of Zn supplementation with different dietary Ca and P recommendations; and (4) determine the supplemental Zn requirement (when utilizing Zn-AA in the diet) for key performance parameters.

## 2. Materials and Methods

### 2.1. Birds and Housing

All animal care procedures and protocols conformed to the South African Poultry Association’s code of practice and were approved by the University of Pretoria Ethics Committee. A total of 5040 mixed-sex, day-old Ross 308 broiler chicks were obtained from a commercial hatchery and assigned to 84 floor pens (60 broilers/pen), with 12 replicate pens per treatment. A randomized block design was used, with every seven consecutive pens containing all seven treatments considered as a block. This was done in order to account for any temperature or spatial variation within the house. Chicks were weighed and assigned to pens in such a way as to ensure minimal BW variation between the pens. Fresh pine wood shavings (5 kg) were weighed into each of the pens at the start of the trial. Pens were in a commercial broiler house where the ambient temperature was initially maintained at 35 °C and then gradually reduced to 24 °C by 28 d of age and maintained for the remainder of the experiment. Lighting followed a schedule of 1 h of dark and 23 h of light for the first 7 d and then 6 h of dark and 18 h of light for the remainder of the trial. Feed and water were provided ad libitum for the duration of the trial. Broilers were checked twice daily, and any mortalities were weighed and recorded.

### 2.2. Experimental Design and Dietary Treatments

Seven dietary treatments were included in this study (Table 1). The first three treatments were supplemented with 90 ppm Zn-S, with treatments 1 and 3 supplemented with 2000 FTU/kg of a novel consensus bacterial 6-phytase variant (Axtra PHY GOLD, Danisco Animal Nutrition & Health (IFF), Oegstgeest, The Netherlands). Treatments 4 to 7 comprised a dose response of incremental doses of Zn in the form of a Zn–amino acid complex (Zn-AA) (Availa Zn, Zinpro Corporation, Eden Prairie, MN, USA), whereby Zn was chelated to a range of amino acids. This was supplemented at 20, 40, 60, and 80 ppm Zn-AA and 2000 FTU/kg phytase in order to maximize phytate hydrolysis. Furthermore, to evaluate the effect of different Ca and P RECs in the study, treatments 1 and 2 followed the standard breed REC [10] whilst treatments 3 to 7 were formulated according to the UMD REC [29,30,31].

Basal diets for the starter, grower, and finisher phases were manufactured (Table 2) in order to ensure that all other nutrients were formulated to the same value within the various growth phases. Each basal diet was then divided by treatment and growth phase and supplemented with the required amounts of limestone, phytase, MCP, and celite according to the treatment specifications. A constant proportion of the basal diet for all treatments within each growth phase was used (Table 3). All treatment diets utilized the same limestone source, with a gross mean diameter (GMD) of 418 µm [32]. The solubility profile of the limestone was 49.9, 62.5, and 79.3% at 5, 15, and 30 min, respectively (Table 4), using the methodology from Kim et al. [33]. The diets were supplemented with celite as an inert filler to balance the nutrient content to obtain the specified Ca and P REC whilst still maintaining isocaloric and isonitrogenous diets across all treatments (Table 3). Phytase and Zn sources were then supplemented in the diets according to their respective treatment formulations. A Ca and P contribution was applied to the dietary treatments that were supplemented with phytase of 0.189% total Ca and digestible P. The diets were fed following either a three-phase feeding program for the breed REC diets of starter, grower, and finisher (d 0–10, 11–24, 25–37) or a five-phase feeding program as specified in the UMD REC of starter, grower 1, grower 2, finisher 1, and finisher 2 (d 0–10, 11–16, 17–24, 25–30, and 31–37). All diets were conditioned and pelleted (pelleting temperature 80 °C; 4 mm) except for the pre-starter, which was crumbled.

### 2.3. Growth Performance

The bodyweight (BW) of the broilers was recorded at placement (d 0) and at the end of each dietary phase (d 10, 16, 24, 30 and 37) and used to calculate BW gain (BWG). Feed was weighed at the start and end of each phase to determine feed intake (FI). These data were used to calculate the feed conversion ratio (FCR) of the broilers for each dietary phase, which was corrected for mortality using mortality weights to obtain the mortality-corrected FCR (mFCR).

### 2.4. Back Scratch Scoring

On d 37, twenty broilers were randomly selected from each pen and assessed for back scratches. This was based on a 4-point scoring system described by [35], where a score of 3 was attributed to the most severe scratches or wounds larger than 1.5 cm, a score of 2 indicated moderate scratches smaller than 1.5 cm long, a score of 1 indicated superficial scratches and a score of 0 described the absence of scratches.

### 2.5. Carcass Characteristics

At d 37, two male broilers close to the pen’s mean BW per pen were selected and weighed to determine the slaughter weight. Birds were stunned by electronarcosis, exsanguinated, and eviscerated to obtain hot carcass weight (HCW). The breasts were manually removed and weighed to calculate breast yield (g) and expressed as a percentage of carcass weight. The dressing percentage was calculated as HCW divided by live weight, expressed as a percentage.

### 2.6. Mineral Excretion

On d 37, all the litter in each pen was removed using shovels and spread onto a large sheet, where it was manually thoroughly homogenized. A representative sample was then collected using a grab sampling technique from multiple points in the pooled litter. Samples were dried at 80 °C for 48 h to determine dry matter content. The dried excreta were subsequently milled through a 0.50 mm screen and analyzed in duplicate for Zn and P. The mineral excretion was expressed per kilogram of BW of the broiler, which was calculated using the total kg live weight (kg) of broilers produced per pen, including mortalities and sampled broilers.

### 2.7. Tibia Mineralization

After the broilers were weighed at each feed changeover on d 10, 16, 24, 30, and 37, four broilers from pen with BW close to the pen’s mean were selected and euthanized via cervical dislocation. The right legs from each broiler were then excised and pooled per pen, then defleshed to isolate the tibia. and the tibias were dried at 80 °C for 72 h in order to obtain a dry tibia weight. Thereafter, the tibias were defatted using refluxing petroleum ether for 16 h following the methodology from [36]. The tibias were then ashed in ceramic crucibles for 8 h at 600 °C in a muffle furnace, following a method similar to [37]. Ash content was expressed as either milligrams per tibia or as a percentage of the dry tibia weight. The ash was then analyzed using ICP technology [38].

### 2.8. Chemical Analysis

Representative samples of all treatment diets were analyzed according to [39]. Samples were analyzed for ash content (method 942.05), crude fiber (method 962.09), and crude protein (method 988.05). Mineral concentrations were determined using inductively coupled plasma atomic emission spectroscopy as per method 935.13 [40]. Phytase was determined according to a modified version of AOAC method 2000.12 [41] where one FTU was defined as the quantity of phytase that released 1 µmol of inorganic orthophosphate from a 0.0051 mol/L sodium phytate substrate per minute at pH 5.5 at 37 °C [42].

### 2.9. Statistical Analysis

Data were analyzed using JMP 16.0 [41] by a one-way ANOVA, with the pen considered as the experimental unit and the block included as a random effect. Treatment means were separated using Tukey’s HSD test [43]. Additionally, regression analysis was performed on the treatments containing Zn-AA to quantify the relationship between Zn dose in the diet and the measured parameter. Regression responses were discussed only where R^2^ > 0.51 [44]. All significant quadratic regressions for various parameters at term were plotted and used to calculate 90% of the asymptote response [45]. Furthermore, planned orthogonal contrasts were performed to compare the inorganic Zn with 90 ppm Zn-S and phytase (treatment 3) with each treatment that was supplemented with Zn in the form of Zn–amino acid (treatments 4–7). These comparisons were specified a priori in order to align with the study objective to determine whether progressively lower inclusion levels of Zn-AA could produce responses comparable to those obtained with the conventional inorganic Zn supplementation strategy under identical dietary specifications. Categorical data were analyzed using a Rao–Scott chi-square analysis to quantify the significance observed in contingency plots. All effects were considered significant at *p* < 0.05.

## 3. Results

### 3.1. Enzyme Recoveries and Analyzed Nutrients

Table 5 shows the formulated and analyzed values for Ca, P, and Zn in the treatment diets for all phases. Dietary analysis showed that all Ca analyses were within 0.08 percentage points of the formulated values. The analyzed P content was no more than 0.05 percentage points different than the formulated values. Basal diets containing no supplemental Zn contained between 23.48 and 27.05 ppm basal Zn (Table 5). These values were subtracted from the total analyzed dietary Zn concentrations to determine supplemental Zn levels. Across treatments, supplemental Zn levels deviated by no more than 15 ppm from formulated values, except for treatment 2 in the grower and finisher phases, which differed by 27.25 and 19.05 ppm, respectively. Diets without supplemented phytase were below the analytical detectable limits for phytase. Diets containing 2000 FTU/kg feed were within 10% of the expected phytase activity.

### 3.2. Growth Performance

Growth performance results are presented in Table 6, Table 7 and Table 8. Supplementation of 2000 FTU/kg phytase to diets formulated according to the breed REC and containing 90 ppm Zn-S increased (*p* < 0.05) bodyweight (BW) at days (d) 10 and 16 compared to the non-supplemented control (328.9 vs. 319.3 g and 685.4 vs. 673.5 g, respectively). This addition of phytase, however, did not affect mFCR at any stage in the trial, nor the BW post d 16.

No differences in BW were reported between birds fed different REC diets (treatments 1 vs. 3) in any of the recorded phases. However, broilers fed diets following the breed REC with 90 ppm Zn-S (treatment 1) resulted in an impaired (*p* < 0.05) mFCR compared to those fed the UMD REC with the same Zn source and inclusion level (treatment 3) on d 16 (1.143 vs. 1.096), 24 (1.234 vs. 1.206), and 37 (1.409 vs. 1.377). No differences in mFCR were reported between broilers that were provided with different treatment diets during the starter phase.

Feeding broilers diets containing less than 60 ppm Zn-AA resulted in reduced (*p* < 0.05) BW compared to those receiving 80 ppm Zn-AA on days 24, 30 and 37. However, in the earlier phases, feeding broilers diets supplemented with less than 80 ppm Zn-AA resulted in a reduction (*p* < 0.05) in BW compared to the other broilers receiving lower doses of Zn-AA. When considering the optimum level of Zn-AA for the lowest mFCR, an inclusion of 60 ppm was shown to be most efficient (*p* < 0.05) at term (1.365), although this was not different to the mFCR of broilers fed 40 or 80 ppm Zn-AA (1.375 and 1.370, respectively), or those fed 90 ppm Zn-S (1.377). Compared to broilers fed 90 ppm Zn-S on the breed REC (treatment 1), supplementing the UMD diet with 80 ppm Zn-AA resulted in a higher (*p* < 0.05) BW on d 10 (338.8 g vs. 328.9 g), 16 (703.2 g vs. 685.4 g), 30 (1956.0 g vs. 1916.8 g), and 37 (2736.7 g vs. 2680.8 g). This benefit in performance was also reported for mFCR, where broilers fed diets formulated on the UMD Ca and P REC with 80 ppm Zn-AA resulted in a lower (*p* < 0.05) mFCR as compared those fed 90 ppm Zn-S on the breed REC on d 16 (1.092 vs. 1.143), 24 (1.205 vs. 1.234), 30 (1.260 vs. 1.286), and 37 (1.370 vs. 1.409). Regression analysis indicated that BW responded quadratically to graded levels of Zn-AA inclusion (treatments 4, 5, 6, and 7) during the starter (*p* < 0.01; R^2^ = 0.58), grower 1 (*p* = 0.01; R^2^ = 0.62), grower 2 (*p* < 0.01; R^2^ = 0.62), finisher 1 (*p* < 0.01; R^2^ = 0.52), and finisher 2 (*p* < 0.01; R^2^ = 0.60) phases. From d 24 onwards, a plateau in the broiler’s response to Zn-AA was noted in BW when supplementing diets with 60 ppm Zn-AA. The FI of the broilers also elicited a quadratic response in the starter (*p* < 0.01; R^2^ = 0.55) and grower 1 (*p* = 0.01, R^2^ = 0.52) phases.

The contrast analysis reported that broilers fed the UMD diet supplemented with 90 ppm of Zn-S (treatment 3) exhibited a lower *(p* < 0.05) BW and higher (*p* < 0.05) FI than those fed 80 ppm of Zn-AA (treatment 7) at d 10, 16, 24, and 30. When formulating diets using 60 ppm Zn-AA, the performance of both BW and mFCR was either similar or improved relative to the 90 ppm UMD Zn-S treatment during all growth phases according to contrast analysis. Further contrasts indicated that the addition of 80 ppm Zn-AA into the UMD diets resulted in a heavier (*p* < 0.05) BW as compared to the broilers that were fed 90 ppm Zn-S on the same diet type on day 16 (683.9 vs. 703.2 g), 24 (1323.0 vs. 1347.5 g), and 30 (1912.9 vs. 1956.0 g), respectively. However, no effect was reported in mFCR. At term, contrast analysis reported that broilers fed 90 ppm of Zn-S showed a higher BW (*p* < 0.05) and lower mFCR (*p* < 0.05) compared to those fed 20 ppm of Zn-AA under the same Ca and P REC.

### 3.3. Back Scratch Scoring

When considering the effect of Zn supplementation strategy on skin integrity, it was noted that broilers fed treatment diets supplemented with Zn-AA, regardless of the dose, exhibited a higher incidence *(p* < 0.05) of score 0 (the absence of back scratches) (Table 9) compared to broilers fed any diet containing Zn-S. Broilers fed diets supplemented with 40 ppm or higher doses of Zn-AA demonstrated a lower (*p* < 0.05) incidence of severe back scratches (score 3) compared to those fed 90 ppm Zn-S with diets formulated to the breed REC. However, there was no additional benefit on reducing the incidence of back scratches when supplementing broiler diets higher than 40 ppm Zn-AA.

### 3.4. Carcass Characteristics

Carcass characteristics are presented in Table 10. No differences (*p* > 0.05) were observed in carcass parameters among broilers fed treatment diets with 90 ppm Zn-S (treatments 1, 2, and 3) regardless of the Ca and P REC or phytase inclusion. Broilers fed 20 ppm Zn-AA resulted in a lower (*p* < 0.05) slaughter weight than those fed 60 ppm Zn-AA and above, as well as a lower (*p* < 0.05) HCW than those supple-mented with 80 ppm Zn-AA. Both slaughter weight and breast yield (in grams and as a percentage of HCW) were maximized between 40 and 80 ppm Zn-AA, with no further increase in HCW noted when supplementing above 60 ppm Zn-AA. Broilers fed 90 ppm Zn-S under the UMD REC (treatment 3) exhibited a higher (*p* < 0.05) slaughter weight and HCW compared to those fed 20 ppm Zn-AA, but similar to broilers supplemented with 40 ppm Zn-AA and above, as highlighted by the contrast analysis. In addition, breast yield (%) was higher (*p* < 0.05) in broilers fed 40 ppm and above of Zn-AA compared to those fed 90 ppm Zn-S (treatment 3). Regression analysis indicated significant (*p* < 0.01) linear and quadratic regressions for slaughter weight, HCW, and breast yield (g) with increasing Zn-AA doses; however, the coefficients of determination were low (R^2^ = 0.18–0.31). No differences (*p* > 0.05) were observed in dressing percentage between any of the treatment means.

### 3.5. Mineral Excretion

Mineral excretion results are presented in Table 11. Phytase supplementation (2000 FTU/kg; treatment 1) reduced (*p* < 0.05) mineral excretion, compared to those supplemented with no phytase (treatment 2) for both P (4.5 vs. 11.0 g/kg BW) and Zn (179.2 vs. 287.5 mg/kg BW). When examining the effect of different RECs on mineral excretion, it was noted that broilers fed the UMD REC excreted less P (*p* < 0.05) compared to those fed diets formulated to the breed REC (3.1 vs. 4.5 g/kg BW). However, broilers on the UMD REC excreted more (*p* < 0.05) Zn compared to those fed the breed REC (219.7 vs. 179.2 mg/kg BW) given the same source and dose of Zn. The use of increasing levels of Zn-AA in the broiler diets resulted in a stepwise linear increase (*p* < 0.05; R^2^ = 0.93) in Zn excretion, while no effect (*p* > 0.05) was observed for P excretion. The use of contrast analysis highlighted the differences between different Zn supplementation strategies, whereby broilers fed any level of Zn-AA resulted in decreased Zn excretion per kg BW compared to those fed 90 ppm Zn-S.

### 3.6. Tibia Mineralization

The tibia ash content from broilers fed different treatment diets is reported in Table 12. Broilers fed diets following the UMD REC exhibited a higher (*p* < 0.05) tibia ash (%) on d 16 compared to those fed the same dose and source of Zn on the breed REC (52.3 vs. 50.8%). No other differences (*p* > 0.05) in mineralization parameters between the different RECs (treatment 1 vs. treatment 3) were reported. The supplementation of phytase in the diet elicited a response for tibia ash only on d 30 where broilers fed diets containing no phytase reported a lower (*p* < 0.01) tibia ash as compared to those fed diets containing 2000 FTU/kg feed (46.7 vs. 48.1%). No differences (*p* > 0.05) in tibia ash (mg/tibia and %) were detected between graded levels of Zn-AA across all growth phases. On d 16, broilers fed 80 ppm Zn-AA resulted in a higher (*p* < 0.05) tibia ash (mg/tibia) than broilers fed 90 ppm Zn-S on the breed REC (661.0 vs. 575.4 mg/tibia). Furthermore, broilers fed any dose of Zn-AA resulted in a higher (*p* < 0.05) tibia ash (%) compared to those fed Zn-S at 90 ppm with the breed Ca and P REC. Additionally, contrast analysis reported that broilers fed any level of Zn-AA resulted in a higher (*p* < 0.05) tibia ash (mg/tibia) than broilers fed diets containing 90 ppm Zn-S (treatment 3) on d 10.

The effects of the treatment diets on the Zn content of the tibias are reported in Table 13. The supplementation of phytase in treatment 1 increased (*p* < 0.05) the Zn content of the ash (mg/kg) on d 10 (421.0 vs. 391.1), d 24 (472.3 vs. 436.2), d 30 (429.3 vs. 379.9), and d 37 (337.1 vs. 297.5) compared to broilers fed diets with no phytase. A similar effect was reported for Zn content in the tibia where broilers fed diets with phytase exhibited an increased (*p* < 0.05) Zn tibia content compared to those fed no phytase on d 10 (12.0 vs. 10.4 mg) and 37 (88.1 vs. 77.9 mg). Broilers fed diets following the UMD REC with 90 ppm Zn-S exhibited a lower (*p* < 0.05) tibia Zn content on d 10 compared to those fed diets following the breed REC (10.6 vs. 12.0 mg), with no differences (*p* > 0.05) observed during later stages. There was no further benefit from increasing the Zn-AA dose to higher than 60 ppm for Zn content in the tibia (mg) on d 10, 16, 30, and 37. No differences (*p* > 0.05) were noted between the graded levels of Zn-AA on tibia Zn concentration (mg/kg) in any of the phases.

The use of contrast analysis further highlights the differences in Zn deposition in the tibia where Zn-AA supplemented at 60 and 80 ppm (treatments 6 and 7, respectively) resulted in higher (*p* < 0.05) tibia Zn content (mg/tibia) on d 10 compared to Zn-S at 90 ppm (treatment 3). However, contrast analysis reported that at d 30, broilers fed 40 ppm and below of Zn-AA elicited a lower (*p* < 0.01) tibia Zn (mg and mg/kg) as compared to those fed 90 ppm Zn-S. Similarly, on d 37, Zn-AA supplemented at 20, 40 and 60 ppm resulted in lower (*p* < 0.05) tibia Zn (mg/kg) compared to treatment 3. This was also reported for the tibia Zn content at term, where broilers supplemented with Zn-AA, irrespective of dose, reported a lower (*p* < 0.05) tibia Zn content (mg/kg) as compared to broilers fed treatment 3 diets.

### 3.7. Asymptote Responses

**Figure 1:**
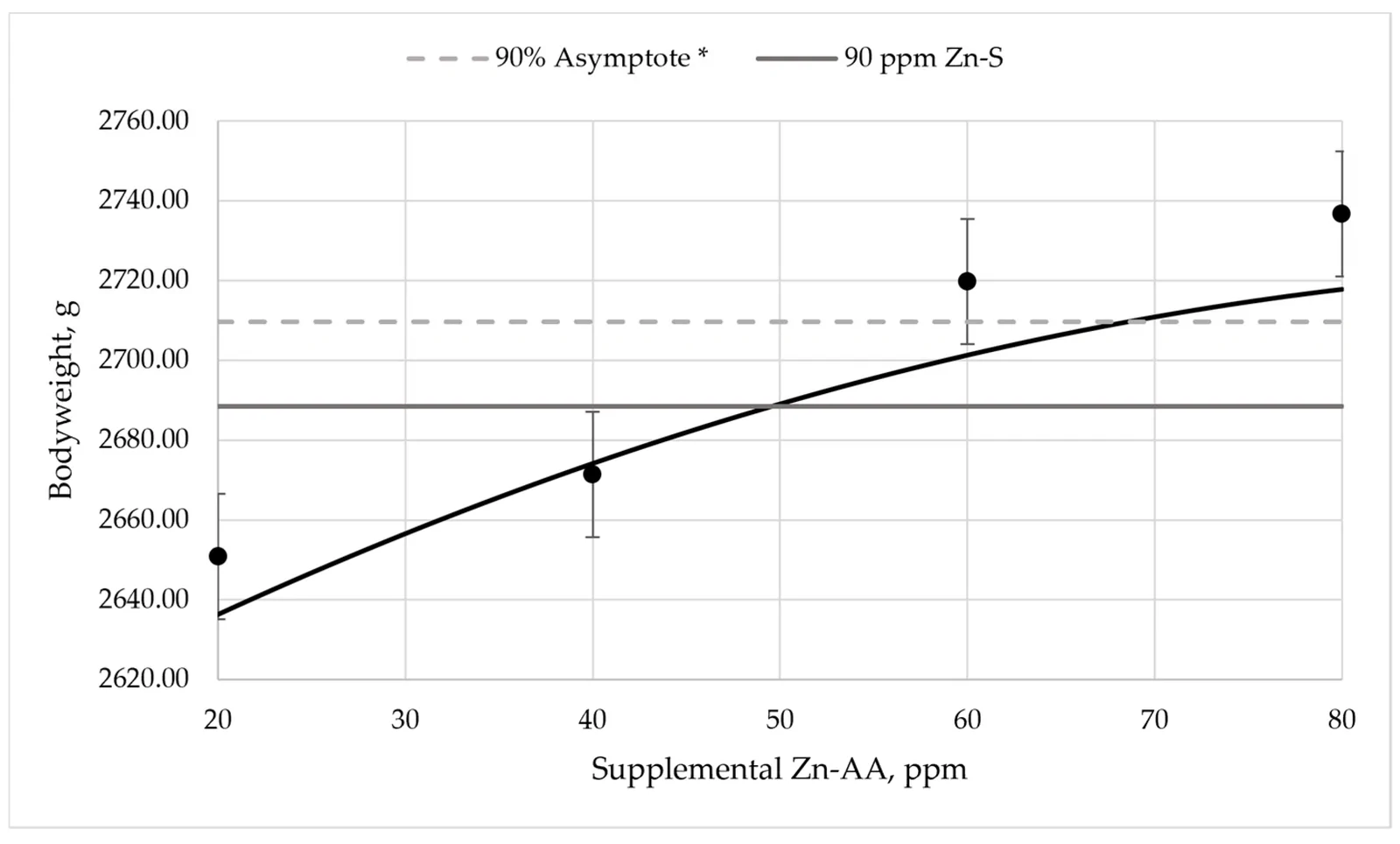
Bodyweight regression response of broilers fed increasing doses of Zn-AA. * Determined using 90% of the broiler response asymptote as described in [45] from diets supplemented with zinc-complexed amino acid (Zn-AA; Availa Zn, Zinpro Corporation, USA) and 2000 FTU/kg of a novel consensus bacterial 6-phytase variant (Axtra PHY GOLD; Danisco Animal Nutrition/IFF, Oegstgeest, The Netherlands).

Based on quadratic regression analysis of the responses to the four Zn-AA supplementation levels, optimum Zn-AA concentrations were estimated for all parameters that exhibited a significant quadratic response (Table 14). The optimum level for each parameter was defined as the Zn-AA concentration corresponding to 90% of the predicted asymptotic response. The levels are presented in Table 13, and they vary between 46 and 68 ppm Zn-AA. The lowest recommendation was for breast yield at 46 ppm of Zn-AA and the highest was for BW at 68 ppm of added Zn-AA (Figure 1).

## 4. Discussion

Currently, the Zn recommendation from the NRC is 40 ppm total dietary Zn [46] and the standard breed recommendation for Zn supplementation in commercial broiler diets is between 100 and 120 ppm [7,8]. However, with increasing use of high doses of phytase, there is the potential to reduce the supplemental levels of Zn in broiler diets. This was noted by Dersjant-Li et al. [27], where broilers fed high phytase doses (1500–2000 FTU/kg) maintained growth performance even in the absence of supplemental trace minerals compared to broilers fed diets with low levels of trace mineral supplementation (up to 30 ppm inorganic Zn and 10 ppm organic Zn). Furthermore, the vast majority of earlier research into the Zn requirements of broilers has predominantly utilized inorganic Zn sources [19,47,48,49] which have been shown to have lower the efficiency of utilization than Zn from organic sources, such as in this study as well as others [5,6,16,17,18]. Certain nutrient recommendations such as that from the Brazilian feed tables even recommend a lower amount of supplemental Zn needed in poultry diets if utilizing Zn from either inorganic (recommended 60 ppm) or organic (recommended 40 ppm) sources [50]. In the present study, Zn complexed to amino acids (Zn-AA) supported equivalent (20–40 ppm) or superior (60–80 ppm) performance compared to 90 ppm Zn-S, indicating a substantial reduction in required supplementation when using organic Zn sources. The differences in bioavailability between Zn sources are likely explained by the difference in absorption mechanisms. Zinc, regardless of the source thereof, is mobilized through ZnT, ZIP, and metallothionine transporter proteins. The functional differences between inorganic and organic sources mainly pertain to absorption dynamics. Research has investigated the absorption pathways of organic forms of Zn such as the one used in this study (Zn chelated with AA) and reported that Zn-AA utilizes AA transporters that are only partially susceptible to Zn uptake antagonists such as Ca and phytic acid [51]. It has also recently been shown that organic Zn enhances Zn absorption in broilers through upregulating Zn ion transporter protein expression in the small intestine [52], as well as mRNA metallothionine levels [25]. The use of such alternative biochemical pathways explains why Zn-AA has a greater bioavailability compared to Zn-S [5,6]. This effect has been quantified in multiple studies that have shown that utilizing organic forms of Zn have resulted in improved (*p* < 0.05) growth or production compared to inorganic sources [17,53,54,55,56,57]. The comparison of inorganic and organic minerals has also been well researched in the literature with regards to bone mineralization in broilers. In the prior literature, the supplementation of organic forms of minerals such as Zn, Cu, and Mn has resulted in increased tibia mineral content as compared to inorganic minerals [58]. In the current study, 90 ppm Zn-S did not maximize tibia Zn deposition during the early growth phases. However, from d24 onwards, broilers fed 90 ppm Zn-S had tibia Zn retention comparable to that of broilers exhibiting the highest tibia Zn retention. From this, we may hypothesize that younger broilers require higher amounts or more bioavailable forms of Zn [26,59] due to the rapid growth and rate of mineralization in the early phases. This has been reported in other studies where the use of graded levels of Zn in the diet elicited a response in Zn content in the tibia on d 21 but not on d 42 [26].

There are other essential roles that Zn plays a fundamental part in including that of maintenance of the integumentary system through the promotion of keratinocytes and modulation of inflammatory cytokine proliferation [60]. Organic Zn supplementation has previously shown to increase the thickness of the dermis layer in broilers compared to those with inorganic Zn supplementation [61]. This may explain the results from the current study, where broilers fed 40 ppm or above of Zn-AA exhibited a decreased (*p* < 0.05) incidence of severe back scratches, which is in agreement with the previous literature [62]. This may be attributed to the combined effects of the role of Zn in the structural integrity of the skin and feathering [1,16,19,61], and the increased bioavailability of organic forms of Zn as compared to inorganic forms [3,4,5,6].

It was also noted in the current study that increasing levels of Zn-AA resulted in a stepwise (*p* < 0.05) increase in slaughter weight, HCW, and breast yield. This may be attributed to the involvement of Zn in the promotion of muscle development through protein synthesis and deposition pathways and myofiber development [63,64,65,66,67]. In particular, research has shown that myoblast proliferation was more pronounced when using Zn chelated to AA compared to inorganic Zn [67]. The effects of different Zn supplementation strategies through the use of different doses and sources of Zn were noted for breast yield percentage in the current study, where 40 ppm and above of Zn-AA resulted in improved breast yield compared to broilers fed 90 ppm Zn-S. This is in agreement with the prior literature [68,69,70,71]. Other studies however have found no effects of different Zn sources on carcass parameters [57,71,72].

The increased bioavailability of organic minerals has also been shown to be a large factor in decreased mineral excretion in broilers [18,55,72,73]. In the current study, the supplementation of 60 ppm and below of Zn-AA decreased Zn excretion whilst still maintaining performance in comparison to broilers fed 90 ppm Zn-S under the same nutritional REC. However, increasing levels of Zn-AA increased Zn excretion in a linear manner. This is due to the homeostatic mechanism of Zn where excess Zn is excreted through gastrointestinal secretion and mucosal cell sloughing, as well as renal excretion [74,75]. These results are in agreement with earlier studies [53,76,77]. In recent years, the EU has called for a decrease in mineral excretion from the poultry sector [9,10]. Although heavy metals are typically a concern, macro minerals such as Ca and P should also be considered. Calcium and P are key nutrients in poultry nutrition, due to their role in the development of the skeletal system as well as in cellular signaling and various essential biochemical processes. However, these minerals can compete for absorption when Ca (particularly in the form of limestone) is supplemented in the diet [78,79]. This interaction is due to the presence of phytic acid or inositol phosphate in the diet which has a strong chelating capacity to divalent cations such as Ca^2+^ and Zn^2+^ [23,80]. This results in decreased Ca and P digestibility due to the formation of insoluble Ca–phytate complexes [79,81]. An imbalance of Ca:P can also have deleterious effects, reducing broiler growth and bone mineralization [82,83]. The combined pressure to reduce excess Ca and P from a performance and mineral sustainability perspective have been key driving factors to refine Ca and P recommendations close to the bird’s requirements, preventing excess nutrient wastage. In this study, the UMD REC contains a higher P content in the form of monocalcium phosphate (MCP) in the earlier dietary phases (d 0 to 10) compared to the breed REC with phytase (0.62% dP vs. 0.53 available P%, respectively; Table 4). This is done with the goal of formulating diets to meet the broiler’s mineralization requirement in the first 16 d which requires more P than that of the broiler’s growth requirement [84,85]. This was noted in the current study where on d 16, broilers fed the UMD REC exhibited a higher tibia ash percentage as compared to broilers fed diets according to the breed REC. From d 16 onwards, the diets following the UMD REC are then formulated to support the broilers’ growth requirements rather than mineralization. It has been reported that an elevated digestible P content in early dietary phases improves growth in young broilers [86]. The higher inclusion of MCP results in the treatment diets containing a smaller amount of limestone due to the relative Ca contribution from MCP. This may further benefit broiler growth due to the reduced dietary acid-binding capacity and subsequent reduced phytic acid chelation observed with limestone sources [87]. This is hypothesized to result in increased Ca digestibility due to the anti-nutritional qualities of Ca from limestone contributing towards enhanced growth [88]. The combined strategy to optimize bone development in the first 16 d, and thereafter prioritize growth, ultimately leads to improved growth performance when coupled with high phytase supplementation. This may explain the broilers’ response in mFCR on d 16, 24, and 37, where broilers fed diets following the UMD REC at 90 ppm Zn-S exhibited a lower mFCR compared to those fed the same source and level of Zn (treatment 1 vs. 3). The combined benefits of using the UMD REC whilst feeding forms of Zn with higher bioavailability were noted at term where broilers fed 60 ppm Zn-AA using the UMD REC had a lower mFCR than other treatment groups. Specifically, their mFCR was 4.4 points lower than broilers fed 90 ppm Zn-S following the breed REC, and 1.2 points lower than broilers fed 90 ppm Zn-S on the same UMD REC. This highlights that using the UMD REC in conjunction with a more bioavailable Zn source resulted in the best improvement in mFCR at term.

It should also be noted that broilers fed 90 ppm Zn-S on the UMD REC exhibited a lower P excretion when compared to broilers fed the same Zn source and dose as those fed diets formulated according to the breed Ca and P REC. This is due to the lower amount of P in the UMD REC in the later growth phases as compared to the more commercially implemented breed REC. However, the broilers fed 90 ppm Zn-S on UMD REC exhibited a higher Zn excretion as compared to those fed the same Zn source and dose on the breed REC. This may be due to interactions with phytate, where, in the UMD REC, the higher dietary Ca concentration combined with a high inclusion of phytase is hypothesized to have altered the mineral–phytate binding dynamics of Zn. Previous studies have demonstrated complex interactions among dietary Ca, Zn, phytate, and phytase. Increasing dietary Ca, primarily through limestone supplementation, promotes the formation of insoluble Ca–phytate complexes as digesta progresses through the upper GIT. These complexes can bind to Zn, forming stable Ca–Zn–phytate complexes that reduce the solubility and availability of Zn for absorption. Furthermore, the precipitation of phytate with Ca limits the accessibility of phytase to the complex, thereby reducing phytate hydrolysis and the subsequent release of Zn and other phytate-bound nutrients [11,14,15,89,90]. Consequently, elevated dietary Ca concentrations may decrease Zn bioavailability through both direct mineral complex formation and indirect inhibition of phytate degradation [51,89,90] and result in increased Zn excretion as reported in the current study. This is in contrast to research conducted by Khaksar et al. [91], where broilers fed diets with high Ca as compared to low Ca in the diets resulted in improved Zn tibia deposition.

The addition of phytase also exhibits a clear benefit in reducing excess mineral excretion [92,93,94] through these mineral dynamics. This was reported in the current study, where the addition of phytase in the diet decreased P and Zn excretion in broilers on a per kg BW basis. This underscores the importance of phytase supplementation in broiler diets, particularly when considering both environmental pollution and the increasing prices of inorganic phosphates. In the current study, the supplementation of phytase in diets following the standard breed REC improved performance in the early phases where diets with 2000 FTU/kg phytase increased (*p* < 0.05) d 10 and 16 BW compared to broilers who received no exogenous phytase. This is most likely due to the extra-phosphoric effects of phytase, where energy and amino acids may be further liberated from the raw materials in the diet [95]. However, the observed response in growth due to phytase supplementation was confined to the early dietary phases. This is hypothesized to be due to the fact that young broilers have an immature digestive tract [96,97] that is more sensitive to the anti-nutritional effects of phytic acid, making phytase supplementation more impactful in the early growth performance of broilers [98,99].

Phytic acid also chelates to a number of divalent cations including Zn, of which IP has the highest chelating capacity [23]. Due to this, phytase supplementation increases the availability of Zn to the broiler [99,100,101] and may increase Zn tibia content [102,103]. This was evidenced in the current study, where phytase supplementation at 2000 FTU/kg increased Zn content in the tibia (mg/tibia and mg/kg) in the early and late stages of growth on d 10, 30, and 37.

Furthermore, the combined effects of Zn source and phytase supplementation were underscored in the early phase broiler mineralization, where on d 10, broilers fed Zn-AA regardless of dose resulted in an increased tibia ash (mg/kg) compared to those fed 90 ppm Zn-S according to contrast analysis. Since Zn forms an extremely small part of the tibia ash itself, one may hypothesize that this is due to the interactive effects between phytase, phytate, and the use of organic Zn.

These biochemical interactions may be supported by the results of the current study, where when supplementing broiler diets with Zn-AA with the UMD Ca and P REC and a high dose of phytase, the optimum responses in broilers were shown to be much lower than the levels recommended by breed companies. When specifically looking at the growth response to increasing Zn-AA supplementation, a quadratic stepwise improvement in BW was seen with increasing Zn-AA levels from d 24 to term. The poor performance associated with lower Zn inclusions may likely be a result of a Zn deficiency in the broilers, particularly when considering the role that Zn plays in growth-related factors such as protein accretion [66], non-specific immunity [4], and GIT development [6,104,105,106]. However, a plateau in d 37 broiler BW was reported when broilers were supplemented with over 60 ppm Zn-AA. The same inclusion level was noted to result in the lowest mFCR at term. In the present study, estimation of the dietary Zn concentration required to achieve 90% of the asymptotic response indicated that the optimal Zn level varied depending on the performance parameter considered. This parameter-specific response aligns with variability in the current literature, where the recommended Zn inclusion levels range between 32 and 180 ppm for optimum growth performance [107]. Such discrepancies among studies for the recommended Zn supplementation level in broilers may be accounted for by multiple factors, including (i) differences in total Zn content in the control diets, (ii) the dose and source of phytase used, (iii) the source of Zn utilized in the diet, as even between organic sources there is large variability, and (iv) the Ca and P levels fed in the diets.

A limitation of the current study is that a full factorial experimental design was not utilized, which would have permitted direct comparisons between the different Zn sources at equivalent supplemental inclusion levels. Future research should consider incorporating a full factorial design to more effectively isolate the independent effects of Zn source and Zn dose, as well as any interactions between these factors, on broiler performance, mineralization, carcass yield, and mineral excretion.

## 5. Conclusions

In the current study, it has been evidenced that broiler performance is influenced by the Zn supplementation strategy utilized (as effected by the source and dose of Zn), as well as phytase inclusion, and dietary Ca and P REC. The supplementation of Zn complexed to amino acids in conjunction with a high dose of phytase sustained or improved broiler growth performance, carcass yield, and skin integrity outcomes at reduced inclusion levels when compared to the widely adopted supplementation of inorganic Zn at 90 ppm. The results from this study highlight the need to reconsider conventional inorganic Zn supplementation strategies, with a shift towards the use of organic Zn sources at lower inclusion levels, particularly when supplementing high doses of phytase in the diet. Such an approach may maintain or improve broiler performance while reducing Zn supplementation and subsequent Zn excretion. Furthermore, it should be noted that the optimal supplemental Zn-AA required to maximize performance is dependent on the parameter being evaluated.

## Figures and Tables

**Table 1:** Trial design.

Treatment	REC ^1^	Zn Source ^2^	Zn, ppm	Phytase ^3^, FTU/kg
T1	Breed REC	Zn-S	90	2000
T2	Breed REC	Zn-S	90	0
T3	UMD	Zn-S	90	2000
T4	UMD	Zn-AA	20	2000
T5	UMD	Zn-AA	40	2000
T6	UMD	Zn-AA	60	2000
T7	UMD	Zn-AA	80	2000

^1^ Recommendations following either the breed [10] or University of Maryland (UMD) [29] Ca and P recommendations. ^2^ Zn supplementation either through zinc sulfate (Zn–S) or a zinc-complexed amino acid (Zn-AA; Availa Zn, Zinpro Corporation, USA). ^3^ 2000 FTU/kg of a novel consensus bacterial 6-phytase variant (Danisco Animal Nutrition/IFF Oegstgeest, The Netherlands).

**Table 2:** Ingredient composition (as-fed basis) of the starter (d 0 to 10), grower (d 11 to 24), and finisher (d 25 to 37) basal diets.

Ingredient, %	Starter	Grower	Finisher
Corn, 7% CP	49.25	52.02	57.82
Soybean meal, 47% CP	33.32	27.49	20.69
Full-fat soybean meal, 36% CP	9.16	12.23	13.32
Sunflower meal, 38% CP	3.06	3.07	3.62
Soybean oil	3.01	3.22	2.60
L-Arg, 98.5%	0.01	0.00	0.03
L-Iso, 90%	0.05	0.03	0.07
L-Thr, 98.5%	0.19	0.16	0.15
L-Val, 98%	0.10	0.07	0.09
L-Met, 99%	0.44	0.40	0.37
L-Lys HCl, 79%	0.32	0.25	0.28
Salt	0.32	0.32	0.32
NaHCO_3_	0.22	0.23	0.23
Vitamin premix ^1^	0.15	0.13	0.13
Zn-free mineral premix ^2^	0.15	0.13	0.13
Choline chloride, 60%	0.20	0.20	0.20
Robenidine ^3^, 6.6%	0.05	0.05	0.05
Total	100.00	100.00	100.00
Formulated nutrients			
ME ^4^, MJ/kg	12.64	13.09	13.20
Crude protein, %	23.45	21.98	19.81
Crude fat, %	7.08	7.85	7.47
dLys	1.32	1.18	1.08
Crude fiber, %	3.36	3.38	3.33
Zinc ^5^, ppm	26.61	27.05	23.48
Phytate P ^6^, %	0.24	0.24	0.24

^1^ Provided per kg of complete diet: Vitamin A from retinol, 12,000 IU; Vitamin D3 from cholecalciferol, 5000 IU; Vitamin E from tocopherol, 60.00 mg; Vitamin K3 from menadione, 2.00 mg; Vitamin B1 from thiamine, 2.00 mg; Vitamin B2, 5.00 mg; Niacin (B3) from nicotinamide, 50.00 mg; Vitamin B5 from pantothenic A, 12.00 mg; Pyridoxine, 3.00 mg; Folic acid, 2.00 mg; Vitamin B12 from cyanocobalar, 0.01 mg; Biotin, 0.10 mg. ^2^ Provided per kg of complete diet: Manganese from manganese sulfate, 100 mg; Iron from iron sulfate, 41.2 mg; Copper from copper sulfate, 10 mg; Cobalt from cobalt sulfate, 0.5 mg; Iodine from calcium iodate, 2.0 mg; Selenium from sodium selenite 0.3 mg. ^3^ Contains 66 g robenidine hydrochloride per kg premix (Zoetis South Africa (Pty) Ltd., Gauteng, South Africa). ^4^ Metabolizable energy (CVB, 2021 [34]). ^5^ Zn from the raw materials in the basal diet. ^6^ Calculated as analyzed phytic acid × 28.18%.

**Table 3:** Ingredient composition of the different treatment diets.

Starter	Treatment 1 ^1^	Treatment 2 ^2^	Treatments 3, 4, 5, 6, 7 ^3^	
Ingredients, %				
Basal diet ^4^	98.04	98.04	98.04	
Limestone	0.34	0.45	0.13	
Celite ^5^	1.38	1.28	1.15	
MCP ^6^	0.23	0.23	0.67	
Phytase	0.0067	0.00	0.0067	
Total	100.00	100.00	100.00	
**Grower**	**Treatment 1**	**Treatment 2**	**Treatments 3, 4, 5, 6, 7 grower 1 ^7^**	**Treatments 3, 4, 5, 6, 7 grower 2 ^8^**
Ingredients, %				
Basal diet	97.76	97.76	97.76	97.76
Limestone	0.14	0.25	0.52	0.47
Celite	1.56	0.46	1.15	1.76
MCP	0.53	1.53	0.56	0.00
Phytase	0.0067	0.00	0.0067	0.0067
Total	100.00	100.00	100.00	100.00
**Finisher**	**Treatment 1**	**Treatment 2**	**Treatments 3, 4, 5, 6, 7 finisher 1 ^9^**	**Treatments 3, 4, 5, 6, 7 finisher 2 ^10^**
Ingredients, %				
Basal diet	98.43	98.43	98.43	98.43
Limestone	0.18	0.28	0.42	0.42
Celite	1.07	0.00	1.14	1.14
MCP	0.31	1.31	0.00	0.00
Phytase	0.0067	0.00	0.0067	0.0067
Total	100.00	100.00	100.00	100.00

^1^ Diets following breed Ca and P recommendations (Aviagen, 2022 [8]) with 2000 FTU/kg added of a novel consensus bacterial 6-phytase variant (30 T Axtra PHY GOLD (contained 42,820 U/g), IFF, Oegstgeest, The Netherlands) (treatment 1).^2^ Diets following breed Ca and P recommendations [10] (treatments 1 and 2). ^3^ Diets following University of Maryland Ca and P recommendations [29] with 2000 FTU/kg added of a novel consensus bacterial 6-phytase variant (30 T Axtra PHY GOLD (contained 42,820 U/g), IFF, Oegstgeest, The Netherlands) (treatments 3, 4, 5, 6, and 7). ^4^ Following a commercial corn-soy diet. ^5^ Inert filler (Celite, Minema Chemicals (Pty) Ltd., Gauteng, South Africa). ^6^ Monocalcium phosphate. ^7^ Treatments 3, 4, 5, 6, and 7 for the grower 1 phase (d 10 to 16). ^8^ Treatments 3, 4, 5, 6, and 7 for the grower 2 phase (d 17 to 24). ^9^ Treatments 3, 4, 5, 6, and 7 for the finisher 1 phase (d 25 to 30). ^10^ Treatments 3, 4, 5, 6, and 7 for the finisher 2 phase (d 31 to 37).

**Table 4:** Characteristics of the limestone used in the trial.

		Solubility ^1^		
		Minutes		
Ca, %	GMD ^2^, µm	5	15	30
37.8	418	49.9	62.5	79.3

^1^ Using a pH 3 glycine buffered solution, as per methodology in Kim et al., (2019) [33]. ^2^ Geometric mean diameter determined using ANSI/ASAE method S319.4 (2008) [32].

**Table 5:** Formulated and analyzed values for the treatment diets.

	Total Ca, %	Analyzable Ca ^1^, %	Total P, %	Available P ^2^, %	dP ^3^, %	Supplemental Zn ^4^, ppm	Phytase ^5^, FTU/kg
Starter							
T1	0.95	0.76 (0.71)	0.53 (0.53)	0.50	0.46	90 (89.70	2000
T2	0.95	0.94 (0.91)	0.74 (0.73)	0.50	0.43	90 (104.3)	0
T3	0.94	0.75 (0.67)	0.62 (0.62)	0.59	0.53	90 (92.1)	2000
T4	0.94	0.75 (0.75)	0.62 (0.61)	0.59	0.53	20 (26.1)	2000
T5	0.94	0.75 (0.73)	0.62 (0.59)	0.59	0.53	40 (44.1)	2000
T6	0.94	0.75 (0.72)	0.62 (0.60)	0.59	0.53	60 (70.3)	2000
T7	0.94	0.75 (0.74)	0.62 (0.64)	0.59	0.53	80 (91.4)	2000
Grower							
T1	0.75	0.56 (0.57)	0.44 (0.46)	0.42	0.39	90 (99.6)	2000
T2	0.75	0.75 (0.81)	0.67 (0.71)	0.42	0.37	90 (117.3)	0
Grower 1							
T3	0.89	0.70 (0.73)	0.45 (0.47)	0.43	0.40	90 (96.9)	2000
T4	0.89	0.70 (0.73)	0.45 (0.48)	0.43	0.40	20 (26.1)	2000
T5	0.89	0.70 (0.67)	0.45 (0.47)	0.43	0.40	40 (44.1)	2000
T6	0.89	0.70 (0.72)	0.45 (0.49)	0.43	0.40	60 (70.3)	2000
T7	0.89	0.70 (0.71)	0.45 (0.49)	0.43	0.40	80 (91.4)	2000
Grower 2							
T3	0.80	0.61 (0.61)	0.34 (0.37)	0.36	0.32	90 (78.2)	2000
T4	0.80	0.61 (0.60)	0.34 (0.37)	0.36	0.32	20 (26.1)	2000
T5	0.80	0.61 (0.62)	0.34 (0.38)	0.36	0.32	40 (44.1)	2000
T6	0.80	0.61 (0.60)	0.34 (0.37)	0.36	0.32	60 (70.3)	2000
T7	0.80	0.61 (0.63)	0.34 (0.37)	0.36	0.32	80 (91.4)	2000
Finisher							
T1	0.65	0.46 (0.48)	0.38 (0.36)	0.36	0.35	90 (91.8)	2000
T2	0.65	0.65 (0.69)	0.58 (0.63)	0.36	0.32	90 (109.1)	0
Finisher 1							
T3	0.69	0.50 (0.52)	0.31 (0.29)	0.30	0.28	90 (80.2)	2000
T4	0.69	0.50 (0.45)	0.31 (0.30)	0.30	0.28	20 (26.1)	2000
T5	0.69	0.50 (0.52)	0.31 (0.29)	0.30	0.28	40 (44.1)	2000
T6	0.69	0.50 (0.50)	0.31 (0.29)	0.30	0.28	60 (70.3)	2000
T7	0.69	0.50 (0.52)	0.31 (0.31)	0.30	0.28	80 (91.4)	2000
Finisher 2							
T3	0.69	0.50 (0.52)	0.31 (0.32)	0.30	0.18	90 (78.0)	2000
T4	0.69	0.50 (0.53)	0.31 (0.32)	0.30	0.18	20 (16.9)	2000
T5	0.69	0.50 (0.45)	0.31 (0.29)	0.30	0.18	40 (33.6)	2000
T6	0.69	0.50 (0.54)	0.31 (0.30)	0.30	0.18	60 (72.3)	2000
T7	0.69	0.50 (0.53)	0.31 (0.31)	0.30	0.18	80 (94.9)	2000

^1^ Defined as the amount of Ca in the diet that can be analyzed chemically (total calcium minus phytase calcium matrix). ^2^ Available P as per [10]. ^3^ Digestible P based on ingredient dP coefficients from [34] and dP from phytase matrix. ^4^ Determined using analyzed Zn minus basal Zn analyses, supplemented either as Zn-S or a Zn-complexed amino acid (Zn-AA; Availa Zn, Zinpro Corporation, USA).^5^ A novel consensus bacterial 6-phytase variant (30 T Axtra PHY GOLD (contained 42,820 U/g), IFF, Oegstgeest, The Netherlands).

**Table 6:** Performance parameters measured during the starter period (d 0 to 10).

0 to 10 d								
Treatment	REC ^1^	Zn Source ^2^	Zn, ppm	Phytase ^3^, FTU/kg	BW, g	FI ^4^, g	mFCR ^5^	Mortality, %
T1	Breed REC	Zn-S	90	2000	328.9 ^b^	294.3 ^ab^	1.027	0.9
T2	Breed REC	Zn-S	90	0	319.3 ^c^	283.8 ^b^	1.013	0.6
T3	UMD	Zn-S	90	2000	324.8 ^bc^	280.3 ^b^	1.004	0.6
T4	UMD	Zn-AA	20	2000	327.6 ^b^	291.6 ^ab^	1.017	1.2
T5	UMD	Zn-AA	40	2000	328.4 ^b^	286.9 ^ab^	1.017	1.1
T6	UMD	Zn-AA	60	2000	329.4 ^b^	290.1 ^ab^	1.011	1.4
T7	UMD	Zn-AA	80	2000	338.8 ^a^	300.3 ^a^	1.012	0.6
Pooled SEM					3.7	3.4	0.006	0.4
*p*-value (ANOVA)					<0.01	<0.01	0.306	0.7
Linear					<0.01	0.03	0.58	0.44
R^2^					0.57	0.45		
Quadratic					<0.01	<0.01	0.83	0.49
R^2^					0.58	0.55		
Contrasts (treatment number)								
3 vs. 4					0.30	0.02	0.08	0.28
3 vs. 5					0.30	0.21	0.28	0.42
3 vs. 6					0.13	0.05	0.22	0.18
3 vs. 7					<0.01	<0.01	0.27	0.99

^a,b,c^ Means within a column with different superscript letters differ (*p* < 0.05). ^1^ Recommendations following either breed [10] or University of Maryland (UMD) [29] Ca and P recommendations. ^2^ Zn supplementation either through zinc sulfate (Zn–S) or a zinc-complexed amino acid (Zn-AA; Availa Zn, Zinpro Corporation, USA). ^3^ 2000 FTU/kg of a novel consensus bacterial 6-phytase variant (Danisco Animal Nutrition/IFF Oegstgeest, The Netherlands). ^4^ Feed intake, g. ^5^ Mortality weight-corrected feed conversion ratio.

**Table 7:** Performance parameters measured until the end of the grower period (d 0 to 16; 0 to 24).

0 to 16 d								
Treatment	REC ^1^	Zn Source ^2^	Zn, ppm	Phytase ^3^, FTU/kg	BW, g	FI ^4^, g	mFCR ^5^	Mortality, %
T1	Breed REC	Zn-S	90	2000	685.4 ^b^	713.2 ^ab^	1.143 ^a^	0.9
T2	Breed REC	Zn-S	90	0	673.5 ^c^	693.1 ^b^	1.134 ^a^	0.6
T3	UMD	Zn-S	90	2000	683.9 ^bc^	693.0 ^ab^	1.096 ^b^	0.6
T4	UMD	Zn-AA	20	2000	692.6 ^b^	708.7 ^ab^	1.089 ^b^	1.8
T5	UMD	Zn-AA	40	2000	687.7 ^b^	699.9 ^ab^	1.084 ^b^	1.5
T6	UMD	Zn-AA	60	2000	691.1 ^b^	704.8 ^ab^	1.086 ^b^	1.7
T7	UMD	Zn-AA	80	2000	703.2 ^a^	724.6 ^a^	1.092 ^b^	0.9
Pooled SEM					4.9	8.7	0.004	0.5
*p*-value (ANOVA)					<0.01	0.04	<0.01	0.26
Linear					0.03	0.03	0.58	0.25
R^2^					0.56	0.37		
Quadratic					0.01	<0.01	0.08	0.65
R^2^					0.62	0.52		
Contrasts (treatment number)								
3 vs. 4					0.13	0.02	0.27	0.06
3 vs. 5					0.35	0.32	0.03	0.15
3 vs. 6					0.21	0.08	0.04	0.10
3 vs. 7					<0.01	<0.01	0.53	0.63
**0 to 24 d**								
T1	Breed REC	Zn-S	90	2000	1331.7 ^abc^	1549.8 ^ab^	1.234 ^a^	1.2
T2	Breed REC	Zn-S	90	0	1314.6 ^c^	1522.1 ^b^	1.228 ^a^	1.1
T3	UMD	Zn-S	90	2000	1323.0 ^bc^	1534.1 ^ab^	1.206 ^bc^	0.8
T4	UMD	Zn-AA	20	2000	1316.3 ^c^	1544.8 ^ab^	1.210 ^b^	2.3
T5	UMD	Zn-AA	40	2000	1318.4 ^c^	1539.3 ^ab^	1.198 ^bc^	2.0
T6	UMD	Zn-AA	60	2000	1335.8 ^ab^	1549.2 ^ab^	1.196 ^c^	2.0
T7	UMD	Zn-AA	80	2000	1347.5 ^a^	1573.4 ^a^	1.205 ^bc^	1.2
Pooled SEM					8.6	12.4	0.005	0.5
*p*-value (ANOVA)					<0.01	0.02	<0.01	0.18
Linear					<0.01	0.02	0.22	0.17
R^2^					0.62	0.04		
Quadratic					<0.01	0.02	0.03	0.66
R^2^					0.62	0.05	0.41	
Contrasts (treatment number)								
3 vs. 4					0.33	0.43	0.53	0.02
3 vs. 5					0.78	0.70	0.44	0.07
3 vs. 6					0.22	0.27	0.16	0.07
3 vs. 7					<0.01	<0.01	0.90	0.49

^a,b,c^ Means within a column with different superscript letters differ (*p* < 0.05). ^1^ Recommendations following either breed [10] or University of Maryland (UMD) [29] Ca and P recommendations. ^2^ Zn supplementation either through zinc sulfate (Zn–S) or a zinc-complexed amino acid (Zn-AA; Availa Zn, Zinpro Corporation, USA). ^3^ 2000 FTU/kg of a novel consensus bacterial 6-phytase variant (Axtra PHY GOLD, Danisco Animal Nutrition/IFF, Oegstgeest, The Netherlands). ^4^ Feed intake, g. ^5^ Mortality weight-corrected feed conversion ratio.

**Table 8:** Performance parameters measured until the end of the finisher period (d 0 to 30; 0 to 37).

0 to 30 d								
Treatment	REC ^1^	Zn Source ^2^	Zn, ppm	Phytase ^3^, FTU/kg	BW, g	FI ^4^, g	mFCR ^5^	Mortality, %
T1	Breed REC	Zn-S	90	2000	1916.8 ^bc^	2378.3 ^ab^	1.286 ^ab^	1.8
T2	Breed REC	Zn-S	90	0	1908.6 ^bc^	2317 ^ab^	1.295 ^a^	1.4
T3	UMD	Zn-S	90	2000	1912.9 ^bc^	2353.9 ^ab^	1.266 ^bc^	1.1
T4	UMD	Zn-AA	20	2000	1894.9 ^c^	2345.7 ^ab^	1.267 ^bc^	2.4
T5	UMD	Zn-AA	40	2000	1908.7 ^bc^	2362.3 ^ab^	1.256 ^c^	2.1
T6	UMD	Zn-AA	60	2000	1933.2 ^ab^	2365.5 ^ab^	1.254 ^c^	2.1
T7	UMD	Zn-AA	80	2000	1956.0 ^a^	2408.0 ^a^	1.260 ^c^	1.7
Pooled SEM					10.7	15.2	0.004	0.5
*p*-value (ANOVA)					<0.01	<0.01	<0.01	0.50
Linear					<0.01	<0.01	0.28	0.36
R^2^					0.51	0.23		
Quadratic					<0.01	<0.01	0.14	0.89
R^2^					0.52	0.22		
Contrasts (treatment number)								
3 vs. 4					0.14	0.70	0.87	0.06
3 vs. 5					0.97	0.69	0.29	0.14
3 vs. 6					0.13	0.57	0.13	0.14
3 vs. 7					<0.01	0.01	0.47	0.40
**0 to 37 d of age**								
T1	Breed REC	Zn-S	90	2000	2680.8 ^bc^	3509.1 ^bc^	1.409 ^a^	1.8
T2	Breed REC	Zn-S	90	0	2685.1 ^bc^	3443.1 ^c^	1.407 ^a^	1.8
T3	UMD	Zn-S	90	2000	2688.4 ^bc^	3593.3 ^ab^	1.377 ^bc^	1.5
T4	UMD	Zn-AA	20	2000	2650.8 ^c^	3556.4 ^ab^	1.385 ^b^	2.6
T5	UMD	Zn-AA	40	2000	2671.4 ^bc^	3582.0 ^ab^	1.375 ^bc^	3.0
T6	UMD	Zn-AA	60	2000	2719.8 ^ab^	3591.5 ^ab^	1.365 ^c^	2.7
T7	UMD	Zn-AA	80	2000	2736.7 ^a^	3655.1 ^a^	1.370 ^bc^	2.0
Pooled SEM					15.7	24.7	0.004	0.6
*p*-value (ANOVA)					<0.01	<0.01	<0.01	0.49
Linear					<0.01	<0.01	0.04	0.36
R^2^					0.59	0.10	0.30	
Quadratic					<0.01	<0.01	0.04	0.48
R^2^					0.60	0.08	0.35	
Contrasts (treatment number)								
3 vs. 4					0.03	0.25	0.03	0.21
3 vs. 5					0.51	0.72	0.50	0.08
3 vs. 6					0.14	0.95	0.14	0.15
3 vs. 7					0.08	0.06	0.08	0.59

^a,b,c^ Means within a column with different superscript letters differ (*p* < 0.05). ^1^ Recommendations following either breed [10] or University of Maryland (UMD) [29] Ca and P recommendations. ^2^ Zn supplementation either through zinc sulfate (Zn–S) or a zinc-complexed amino acid (Zn-AA; Availa Zn, Zinpro Corporation, USA). ^3^ 2000 FTU/kg of a novel consensus bacterial 6-phytase variant (Axtra PHY GOLD, Danisco Animal Nutrition/IFF, Oegstgeest, The Netherlands). ^4^ Feed intake g. ^5^ Mortality weight-corrected feed conversion ratio.

**Table 9:** Percentage of back scratch scores for different treatments evaluated at d 37.

Treatment	REC ^1^	Zn Source ^2^	Zn, ppm	Phytase ^3^, FTU/kg	0	1	2	3
T1	Breed REC	Zn-S	90	2000	5.0 ^b^	47.5 ^ab^	35.4 ^a^	12.1 ^ab^
T2	Breed REC	Zn-S	90	0	3.8 ^b^	39.2 ^b^	33.3 ^a^	23.8 ^a^
T3	UMD	Zn-S	90	2000	4.6 ^b^	45.0 ^ab^	31.7 ^a^	18.8 ^a^
T4	UMD	Zn-AA	20	2000	15.9 ^a^	52.7 ^a^	18.6 ^b^	12.7 ^ab^
T5	UMD	Zn-AA	40	2000	11.7 ^a^	56.3 ^a^	25.0 ^b^	7.1 ^b^
T6	UMD	Zn-AA	60	2000	14.6 ^a^	55.4 ^a^	24.6 ^b^	5.4 ^b^
T7	UMD	Zn-AA	80	2000	15.0 ^a^	49.4 ^a^	26.6 ^ab^	9.0 ^b^
*p*-value (ANOVA)					0.05			

^a,b^ Means within a column with different superscript letters differ (*p* < 0.05). ^1^ Recommendations following either breed [10] or University of Maryland [29] Ca and P recommendations. ^2^ Zn supplementation either through zinc sulfate (Zn–S) or a zinc-complexed amino acid (Zn-AA; Availa Zn, Zinpro Corporation, USA). ^3^ 2000 FTU/kg of a novel consensus bacterial 6-phytase variant (Axtra PHY GOLD, Danisco Animal Nutrition/IFF, Oegstgeest, The Netherlands).

**Table 10:** Carcass yield of the broilers processed at d 37.

Treatment	REC ^1^	Zn Source ^2^	Zn Level ppm	Phytase ^3^, FTU/kg	Slaughter Weight, g	Hot Carcass Weight ^4^, g	Dressing ^5^, %	Breast Yield, g	Breast, %
T1	Breed REC	Zn-S	90	2000	2775.7 ^ab^	2209.9 ^ab^	79.7	647.3 ^ab^	29.3 ^ab^
T2	Breed REC	Zn-S	90	0	2776.8 ^ab^	2218.8 ^ab^	79.9	667.0 ^ab^	30.0 ^ab^
T3	UMD	Zn-S	90	2000	2806.6 ^a^	2230.8 ^ab^	79.6	653.4 ^ab^	29.3 ^b^
T4	UMD	Zn-AA	20	2000	2739.9 ^b^	2182.5 ^b^	79.7	636.4 ^b^	29.2 ^b^
T5	UMD	Zn-AA	40	2000	2775.7 ^ab^	2197.0 ^b^	79.2	673.4 ^a^	30.3 ^a^
T6	UMD	Zn-AA	60	2000	2804.9 ^a^	2236.4 ^ab^	79.7	678.0 ^a^	30.3 ^a^
T7	UMD	Zn-AA	80	2000	2810.0 ^a^	2254.1 ^a^	80.2	672.5 ^a^	30.1 ^a^
Pooled SEM					15.6	14.1	0.4	9.8	0.4
*p*-value (ANOVA)					0.01	<0.01	0.41	<0.01	<0.01
Linear					<0.01	<0.01	0.43	<0.01	<0.01
R^2^					0.18	0.29		0.22	0.04
Quadratic					<0.01	<0.01	0.43	<0.01	0.06
R^2^					0.21	0.31		0.26	
Contrasts (treatment number)									
3 vs. 4					0.01	0.01	0.95	0.17	0.77
3 vs. 5					0.15	0.06	0.49	0.56	0.01
3 vs. 6					0.53	0.38	0.90	0.36	0.02
3 vs. 7					0.77	0.52	0.41	0.33	0.04

^a,b^ Means within a column with different superscript letters differ (*p* < 0.05).^1^ Recommendations following either breed [10] or University of Maryland [35] Ca and P recommendations. ^2^ Zn supplementation either through zinc sulfate (Zn–S) or a zinc-complexed amino acid (Zn-AA; Availa Zn, Zinpro Corporation, USA). ^3^ 2000 FTU/kg of a novel consensus bacterial 6-phytase variant (Axtra PHY GOLD; Danisco Animal Nutrition/IFF, Oegstgeest, The Netherlands). ^4^ Calculated from slaughter weight after evisceration. ^5^ Calculated as hot carcass weight divided by slaughter weight expressed as a percentage.

**Table 11:** Mineral excretion from broilers fed various treatment diets.

Treatment	REC ^1^	Zn Source ^2^	Zn Level ppm	Phytase ^3^ FTU/kg	P Excretion, g/kg BW	Zn Excretion, mg/ kg BW
T1	Breed REC	Zn-S	90	2000	4.5 ^b^	179.2 ^c^
T2	Breed REC	Zn-S	90	0	11.0 ^a^	287.5 ^a^
T3	UMD	Zn-S	90	2000	3.1 ^c^	219.7 ^b^
T4	UMD	Zn-AA	20	2000	3.0 ^c^	81.2 ^f^
T5	UMD	Zn-AA	40	2000	2.9 ^c^	117.6 ^e^
T6	UMD	Zn-AA	60	2000	2.9 ^c^	156.1 ^d^
T7	UMD	Zn-AA	80	2000	2.9 ^c^	191.4 ^c^
Pooled SEM					1.5	4.2
*p*-value (ANOVA)					<0.01	<0.01
Linear					0.33	<0.01
R^2^						0.93
Quadratic					0.75	0.49
R^2^						
Contrasts (treatment number)						
3 vs. 4					0.74	<0.01
3 vs. 5					0.41	<0.01
3 vs. 6					0.42	<0.01
3 vs. 7					0.31	<0.01

^a,b,c,d,e,f^ Means within a column with different superscript letters differ (*p* < 0.05).^1^ Recommendations following either breed [10] or University of Maryland [35] Ca and P recommendations. ^2^ Zn supplementation either through zinc sulfate (Zn–S) or a zinc-complexed amino acid (Zn-AA; Availa Zn, Zinpro Corporation, USA). ^3^ 2000 FTU/kg of a novel consensus bacterial 6-phytase variant (Axtra PHY GOLD; Danisco Animal Nutrition/IFF, Oegstgeest, The Netherlands).

**Table 12:** Tibia mineralization of broilers fed various treatment diets.

					d 10		d 16		d 24		d 30		d 37	
Treatment	REC ^1^	Zn Source ^2^	Zn Level ppm	Phytase ^3^ FTU/kg	Tibia Ash, mg/Tibia	Tibia Ash, %	Tibia Ash, mg/Tibia	Tibia ash, %	Tibia Ash, mg/Tibia	Tibia ash, %	Tibia Ash, mg/Tibia	Tibia Ash, %	Tibia Ash, mg/Tibia	Tibia Ash, %
T1	Breed REC	Zn-S	90	2000	265.3 ^ab^	51.0	575.4 ^b^	50.8 ^c^	1173.6	50.3	1658.4	48.1 ^a^	2517.9	50.2
T2	Breed REC	Zn-S	90	0	284.3 ^ab^	50.8	576.9 ^b^	51.1 ^bc^	1171.2	50.8	1607.6	46.7 ^b^	2600.3	50.9
T3	UMD	Zn-S	90	2000	258.5 ^b^	50.4	606.7 ^ab^	52.3 ^a^	1142.6	49.9	1658.9	47.6 ^ab^	2667.5	50.4
T4	UMD	Zn-AA	20	2000	283.6 ^ab^	50.8	622.1 ^ab^	52.1 ^a^	1176.1	50.0	1645.4	46.8 ^b^	2638.4	50.4
T5	UMD	Zn-AA	40	2000	275.3 ^ab^	50.6	608.3 ^ab^	51.9 ^ab^	1177.8	50.5	1641.1	47.4 ^ab^	2579.2	50.1
T6	UMD	Zn-AA	60	2000	278.0 ^ab^	51.8	612.4 ^ab^	52.5 ^a^	1180.2	50.4	1674.8	47.5 ^ab^	2600.5	50.2
T7	UMD	Zn-AA	80	2000	285.3 ^a^	51.3	661.0 ^a^	52.2 ^a^	1196.0	50.1	1670.7	47.7 ^ab^	2657.2	50.2
Pooled SEM					21.5	0.4	14.6	0.2	27.5	0.3	26.0	0.3	65.6	0.2
*p*-value (ANOVA)					<0.01	0.28	<0.01	<0.01	0.60	0.24	0.58	<0.01	0.78	0.13
Linear					0.73	0.18	0.05	0.31	0.65	0.84	0.32	0.03	0.72	0.29
R^2^							0.08					0.26		
Quadratic					0.63	0.38	0.01	0.53	0.88	0.27	0.62	0.07	0.50	0.57
R^2^							0.18							
Contrasts (treatment number)														
3 vs. 4					>0.01	0.47	0.46	0.45	0.39	0.99	0.13	0.03	0.77	0.83
3 vs. 5					0.04	0.70	0.94	0.20	0.37	0.16	0.63	0.67	0.38	0.37
3 vs. 6					>0.01	0.02	0.79	0.86	0.33	0.23	0.67	0.83	0.51	0.48
3 vs. 7					>0.01	0.13	>0.01	0.92	0.28	0.86	0.75	0.82	0.92	0.69

^a,b,c^ Means within a column with different superscript letters differ (*p* < 0.05). ^1^ Recommendations following either breed [10] or University of Maryland [29] Ca and P recommendations. ^2^ Zn supplementation either through zinc sulfate (Zn–S) or a zinc-complexed amino acid (Zn-AA; Availa Zn, Zinpro Corporation, USA). ^3^ 2000 FTU/kg of a novel consensus bacterial 6-phytase variant (Axtra PHY GOLD; Danisco Animal Nutrition/IFF, Oegstgeest, The Netherlands).

**Table 13:** Zn content and concentration in the tibia of broilers fed various treatment diets.

					d 10		d 16		d 24		d 30		d 37	
Treatment	REC ^1^	Zn Source ^2^	Zn Level ppm	Phytase ^3^ FTU/kg	Zn in Tibia, mg	Zn, mg/kg	Zn in Tibia, mg	Zn, mg/kg	Zn in Tibia, mg	Zn, mg/kg	Zn in Tibia, mg	Zn, mg/kg	Zn in Tibia, mg	Zn, mg/kg
T1	Breed REC	Zn-S	90	2000	12.0 ^a^	421.0 ^a^	24.9 ^b^	433.5	56.4 ^ab^	472.3 ^a^	68.4 ^abcd^	429.3 ^a^	88.1 ^ab^	337.1 ^a^
T2	Breed REC	Zn-S	90	0	10.4 ^c^	391.1 ^b^	25.7 ^b^	447.7	51.7 ^b^	436.2 ^b^	62.9 ^d^	379.9 ^c^	77.9 ^c^	297.5 ^c^
T3	UMD	Zn-S	90	2000	10.6 ^bc^	410.22 ^ab^	25.5 ^b^	430.8	54.3 ^ab^	470.5 ^a^	71.3 ^a^	425.3 ^a^	92.6 ^a^	340.0 ^a^
T4	UMD	Zn-AA	20	2000	11.1 ^abc^	401.8 ^ab^	26.7 ^b^	423.0	52.2 ^ab^	450.6 ^ab^	65.2 ^bcd^	395.0 ^bc^	83.2 ^bc^	315.3 ^b^
T5	UMD	Zn-AA	40	2000	11.3 ^abc^	411.6 ^ab^	26.1 ^b^	431.4	55.2 ^ab^	462.7 ^ab^	64.7 ^cd^	394.2 ^bc^	84.2 ^abc^	319.5 ^b^
T6	UMD	Zn-AA	60	2000	11.8 ^ab^	415.9 ^a^	26.7 ^ab^	428.4	53.7 ^ab^	459.6 ^ab^	70.5 ^ab^	420.7 ^ab^	86.2 ^ab^	327.8 ^ab^
T7	UMD	Zn-AA	80	2000	12.0 ^a^	420.8 ^a^	28.7 ^a^	431.7	57.6 ^a^	470.5 ^a^	69.2 ^abc^	413.9 ^ab^	87.0 ^ab^	327.2 ^ab^
Pooled SEM					0.4	6.4	0.7	9.5	1.4	7.5	1.4	7.0	2.1	4.5
*p*-value (ANOVA)					<0.01	<0.01	0.01	0.69	0.04	<0.01	<0.01	<0.01	<0.01	<0.01
Linear					0.02	0.02	0.04	0.89	0.04	0.14	<0.01	<0.01	0.16	0.03
R^2^					0.12	0.38	0.67		0.22		0.33	0.22		0.33
Quadratic					0.08	0.67	0.10	0.68	0.06	0.11	0.04	0.03	0.51	0.08
R^2^											0.32	0.22		
Contrasts (treatment number)														
3 vs. 4					0.26	0.28	0.22	0.94	0.30	0.04	<0.01	<0.01	<0.01	<0.01
3 vs. 5					0.09	0.85	0.53	0.96	0.68	0.43	<0.01	<0.01	<0.01	<0.01
3 vs. 6					<0.01	0.46	0.24	0.82	0.75	0.26	0.67	0.89	0.03	0.03
3 vs. 7					<0.01	0.17	>0.01	0.94	0.11	0.99	0.26	0.39	0.06	0.03

^a,b,c,d^ Means within a column with different superscript letters differ (*p* < 0.05). ^1^ Recommendations following either breed [10] or University of Maryland [29] Ca and P recommendations. ^2^ Zn supplementation either through zinc sulfate (Zn–S) or a zinc-complexed amino acid (Zn-AA; Availa Zn, Zinpro Corporation, USA). ^3^ 2000 FTU/kg of a novel consensus bacterial 6-phytase variant (Axtra PHY GOLD; Danisco Animal Nutrition/IFF, Oegstgeest, The Netherlands).

**Table 14:** Recommended supplemental level of Zn-AA based on quadratic regressions for various parameters at d 37.

Parameter	Supplemental Recommended Zn Level ^1^, ppm
BW, g	68
mFCR ^2^	49
Slaughter weight, g	62
Hot carcass weight ^3^, g	66
Breast yield, g	46

^1^ Determined using 90% of the broiler response asymptote as described in [45] from diets supplemented with zinc-complexed amino acid (Zn-AA; Availa Zn, Zinpro Corporation, USA) and 2000 FTU/kg of a novel consensus bacterial 6-phytase variant (Axtra PHY GOLD; Danisco Animal Nutrition/IFF, Oegstgeest, The Netherlands). ^2^ Mortality weight-corrected feed conversion ratio. ^3^ Calculated from the slaughter weight after evisceration.

## Data Availability

Data are unavailable due to privacy restrictions by the institutions.

## Abbreviations

The following abbreviations are used in this manuscript:

Zn-AA	Zinc amino acid complex
Zn-S	Zinc sulfate
Ca	Calcium
P	Phosphorus
REC	Recommendation
UMD	University of Maryland
d	Day
BW	Bodyweight
mFCR	Mortality-corrected feed conversion ratio
HCW	Hot carcass weight
CP	Crude protein
